# Mallampati score in patients with temporomandibular joint disorders: A pilot case‐control study

**DOI:** 10.1002/cre2.866

**Published:** 2024-03-03

**Authors:** Amirtaher Mirmortazavi, Azam Sadat Madani, Saeed Hassanzadeh, Reza Shakiba

**Affiliations:** ^1^ Department of Prosthodontics, School of Dentistry Mashhad University of Medical Sciences Mashhad Iran; ^2^ Faculty of Dentistry Mashhad University of Medical Sciences Mashhad Iran

**Keywords:** Mallampati score, PSQI, STOP‐BANG, temporomandibular joint disorders

## Abstract

**Objectives:**

Temporomandibular joint disorder (TMD) is defined as any functional abnormalities in different parts of the face and neck. The Mallampati index is an indicator for determining the extent of airway blockage. No study has examined the relationship between TMD and Mallampati score. Most studies have investigated the relationship between temporomandibular joint problems and sleep problems. This pilot study aimed to assess the Mallampati index scores among TMD patients.

**Material and Methods:**

Eighty‐four people were divided into the case (based on RDC/TMD) and control groups. Demographic information, neck circumference, tongue size, Mallampati score, and other variables were asked of people. STOP‐BANG and Pittsburgh Sleep Quality Index (PSQI) were also completed for each patient. Data were analyzed with Chi‐square, Fisher's exact, and Mann–Whitney tests.

**Results:**

The Mallampati and PSQI questionnaire scores in the case group were significantly higher than those in the control group (*p* < 0.001). The results showed that larger tongue and neck circumference patients had a higher Mallampati score. Pearson correlation coefficient showed that the Mallampati score had a direct and significant relationship with body mass index and PSQI (*p* < 0.001).

**Conclusions:**

The results of this study show that Mallampati scores were significantly higher among patients with TMD than among healthy individuals.

## 1 INTRODUCTION

The temporomandibular joint is classified as a ginglymoarthrodial joint. This means that it is joint with the ability to move hinged and slip along with the bone component that is covered and attached to it by a fibrosis capsule (Burket et al., 2003). Temporomandibular joint disorder (TMD) has been defined as the presence of any functional abnormalities in different parts of the face and neck that have specific clinical symptoms such as pain, limitation in movement and mandibular operation, and sound from the temporomandibular joint. TMD is a common term for all the pain and dysfunctions in the neck, face, mouth, and generally the masticatory system, which can show its symptoms differently (Cuccia et al., 2007).

Numerous causes for TMD have been suggested, including parafunctional habits such as gnashing and clenching teeth, biting the lip, macro or micro trauma, orthopedic instability, joint weakness and laxity, and musculoskeletal and rheumatologic disease (Veasey & Rosen, 2019). The prevalence of TMD, its etiologic factors, signs, and clinical symptoms in different societies vary according to age, race, geographical location, and study time (Tabatabaian et al., 2013). According to the National Institute of Dental and Craniofacial Research, the prevalence of temporomandibular disorders ranges from 5% to 12% (Frohnhofen, 2021).

It is commonly reported that TMD is more prevalent in women than men. Women are 1.5−2 times more likely than men to develop this condition, ascribed to behavioral, hormonal, anatomical, and psychological variables. While some research studies indicate that TMD is more prevalent in adults, several indicate a similar prevalence in younger people and adults (Ferendiuk et al., 2014).

The studies have reported the relationship between TMD and obstructive sleep apnea (OSA) (Mannarino et al., 2012). OSA is a chronic disorder marked by upper airway collapse during sleep. OSA can occur at all stages of sleep but is more common in the Rapid Eye Movement (REM) stage. OSA has persistent snoring, daytime sleepiness, fatigue, and hypoxia. Complications of this disorder include high blood pressure, cardiovascular disease, and abnormal glucose metabolism. Increased daily sleep, cognitive disorder, impaired work, anxiety, and problems in personal relationships are other complications of this disorder (Veasey & Rosen, 2019). The Mallampati index (*M* score) is one of the criteria that can be applied to diagnose and predict OSA based on measuring the distance from the soft palate to the base of the tongue (Ferendiuk et al., 2014).

The Mallampati index is an indicator for determining the extent of airway blockage. Sleep apnea is related to a reduction in upper airway size, which is more common in those who are obese or have anatomical abnormalities such as larger tongues or tonsils (Sönmez et al., 2001).

For more than 20 years, the Mallampati score has been used to recognize patients at threat for difficult tracheal intubation because of its noninvasive ways and uncomplicated learning, necessitating no specific equipment (Samsoon & Young, 1987).

No study has examined the relationship between TMD and Mallampati score. Most studies have investigated the relationship between TMD and sleep problems (Amra et al., 2019; Tabatabaian et al., 2013). To respond to this knowledge gap, this study aimed to evaluate the Mallampati index scores among TMD patients.

## 2 MATERIALS AND METHODS

### 2.1 Study design and ethical considerations

This pilot case‐control study was performed on patients referred to the Prosthodontics Department of Mashhad Dental School between 2019 and 2020. In this study, STROBE guideline policies have been followed to the extent possible (Von Elm et al., 2007). Written consent was obtained from all patients who participated in this study. All Helsinki Declarations were followed and the study obtained the ethics code of IR.MUMS.DENTISTRY.REC.1399.033 from the ethics committee of the Mashhad University of medical science.

### 2.2 Participants

Patients over 18 years old and under 60 years old who had no history of trauma to the joints, face, and neck in the last 3 months and no complications in the face, oral, and dental examinations that would cause similar symptoms in the temporomandibular joint areas were included in this study. Exclusion criteria included uncontrolled systemic disease, neurological disorders, head, and neck cancer, edentulous patients, patients with a history of TMJ surgery, and patients with idiopathic clinical symptoms.

After screening by the eligibility criteria, the participants were divided into two groups, case and control, based on TMD diagnosis:

Case group: all patients whose TMD was diagnosed by an experienced specialist based on RDC/TMD with RDC I or RDC III classification (Wright, 2005).

Control group: intact subjects of the study, who did not meet the exclusion criteria, participated in the study as a control group.

### 2.3 Data acquisition

Demographic information of patients including age, sex, neck circumference (Above 40 cm were considered as high neck circumference), body mass index (BMI) index, duration of joint disease, type of joint disease, tongue size (The large tongue or macroglossia was diagnosed with examinations such as tongue sticking out of the mouth, difficulty speaking, frequent traumas to the tongue, and abnormal growths of the mouth, jaw, and face [Topouzelis et al., 2011]) and patient's chief complaint that made them referred to our Prosthodontics department was recorded separately for each individual. In addition, tooth wear, pain, bruxism, pressure teeth, hands under the chin, playing musical instruments (musical instruments related to temporomandibular joint involvement), object biting, cheek biting, and chewing gum by questionnaires from Case and control groups were asked.

### 2.4 Measuring the mallampati score

The same clinician performed a clinical examination of patients to determine the Mallampati index. To determine the Mallampati index, the patient is asked to protrude his tongue during the maximum opening of the mouth. The degree of upper airway obstruction and Mallampati index are defined according to how much the tonsils, uvula, and soft palate can be seen (Ferendiuk et al., 2014). Mallampati score is classified into four classes (Tham et al., 1992):

Class I: Soft palate, uvula, throat, and tonsillar columns are seen.
Class II: Soft palate, uvula, and throat are seen.
Class III: Soft palate and uvula base are seen.
Class IV: only the hard palate is seen.

**Figure 1:**
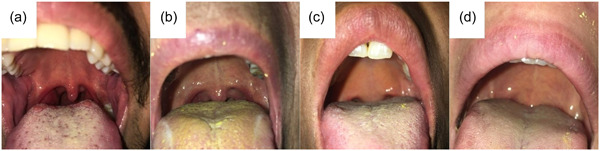
Mallampati index classification; (a): Class I, (b): Class II, (c): Class III, (d): Class IV.

Photographs of patients with different Mallampati scores are shown below (Figure 1).

### 2.5 Sleep monitoring

Two questionnaires were asked of the study's participants to monitor their sleep. STOP‐BANG sleep apnea questionnaire includes eight questions about male gender, history of frequent snoring loudly in sleep, daytime drowsiness, sleep apnea, history of hypertension, age over 50 years, BMI of 35 kg/m^2^, and a neck circumference over 40 cm, which was yes (1) or no (0) question to screen for the possibility of OSA. The score range is between zero and 8 points. A score of 1−2 indicates a low probability, 3−4 means moderate probability, and five or higher indicates a high probability of OSA (Amra et al., 2018).

The Pittsburgh Sleep Quality Index (PSQI) has seven scales that include mental quality of sleep, delay in falling asleep, duration of proper sleep, adequacy of sleep (the ratio of appropriate sleep time to time spent in bed), sleep disorders (waking up at night), the dosage of intake sleeping pills, and dysfunction of daily functioning (problems caused by insomnia during the day). The score of each scale is between 0 and 3, and the score of 3 on each scale indicates the maximum negative. The range score of this questionnaire is 0−21, and the overall score of 6 and above shows inadequate sleep quality. The reliability and validity of this questionnaire have been confirmed in various studies (Scarlata et al., 2013).

### 2.6 Statistical analysis

In this study, the data were analyzed using SPSS software version 25. Data were described using appropriate statistical tables, and Chi‐square, Fisher's exact, and Mann−Whitney *U* tests were used to analyze the data. The significance level in statistical tests was considered equal to 5% (*p* < 0.05).

## 3 RESULTS

In this study, 84 people, including 42 women (50%) and 42 men (50%) with a mean age of 35.20 ± 11.59 years and an age range of 18 to 60 years, participated. These individuals were evaluated in two groups of 42 cases and controls. The mean age was 35.10 ± 11.53 years in the control group and 35.31 ± 11.79 years in the case group. There was no significant difference between the two groups regarding age (*p* = 0.932). In the control and case groups, 21 (50%) were male, and in the control and case groups, 21 (50%) were female. The number of men and women in the control and case groups was quite similar (*p* = 1.00). The mean BMI in the control group was higher than in the case group, but the difference was insignificant (*p* = 0.956). The mean rate of mouth opening in the control group was significantly higher than in the case group (*p* = 0.001) (Table 1).

**Table 1:** Demographic information.

Variable	Case (Mean ± SD)/*n* (%)	Control (Mean ± SD)/*n* (%)	*p* Value
Age (year)	35.31 ± 11.79	35.1 ± 11.53	0.932^a^
Gender			
Male	21 (50%)	21 (50%)	1^b^
Female	21 (50%)	21 (50%)	
Body Mass Index (kg/m^2^)	23.79 ± 3.13	23.82 ± 2.55	0.956^a^
Mouth opening (mm)	43.12 ± 7.20	47.69 ± 5.36	0.001^a^

^a^ Independent *T*‐test.

^b^ Chi‐square test.

Table 2 compares the STOP‐BANG, *M* Score, and PSQI variables between the case and control groups. As can be seen, the range of observations of the STOP‐BANG variable in the case group was less than in the control group. The mean STOP‐BANG in the control group was lower than the case group, and the two groups did not differ significantly in the STOP‐BANG score (*p* = 0.170). The *M* score range was also higher in the case group than in the control group. The mean *M* score in the control group was significantly lower than in the case group (*p* = 0.007). The range of PSQI scores was also higher in the case group than in the control group. The mean PSQI score in the control group was significantly lower than in the case group (*p* = 0.043) (Table 2).

**Table 2:** Comparison of STOP‐BANG, Mallampati score (*M* score), and PSQI between case and control group.

Variable	Case (Mean [min−max])	Control (Mean [min−max])	*p* Value^a^ (z)
STOP‐BANG score	1.74 (0−4)	1.45 (0−5)	0.17 (1.37)
*M* Score	2.38 (1−4)	1.79 (1−3)	0.007 (2.71)
Pittsburgh Sleep Quality Index (PSQI)	6.21 (1−16)	4.86 (1−13)	0.043 (2.02)

^a^ Mann–Whitney *U* test.

Table 3 shows a comparison of neck circumference, tooth wear, joint pain, tongue size, bruxism, pressure teeth, and placement of the hand under the chin in the control and case groups. As can be seen, two cases of high neck circumference were observed in each of the two groups, which was not statistically significant (*p* = 1.00). In the case group, 11.9% and the control group, 7.1% had tooth wear. There was no significant difference between the two groups regarding wear (*p* = 0.713). 54.8% (*n* = 23) had pain in the case group and 2.4% (*n* = 1) in the control group. The amount of pain in the control group was significantly lower than the case group (*p* < 0.001) (The pain that the control group had was muscular and was not caused by TMD). 19% in the case group and 9.5% in the control group had large tongue sizes. The number of large language sizes between the two groups was not statistically significant (*p* = 0.212). In the case group, 28.6% and in the control group, 16.7% had gritted teeth. There was no significant difference between the two groups regarding the number of gritted teeth (*p* = 0.192). In the case group, 45.2%, and in the control group, 19% had pressure teeth. The number of pressure teeth in the case group was significantly lower than in the control group (*p* = 0.010). In the case group, 14.3% and the control group, 38.1%, put their hands under the chin. The number of patients who put their hands under the chin in the control group was significantly higher than the case group (*p* = 0.013) (Table 3).

**Table 3:** Comparison of neck circumference, tooth wear, joint pain, tongue size, bruxism, pressure teeth, and placement of the hand under the chin in the control and case groups.

Variable	Case *n* (%)	Control *n* (%)	*p* Value
High neck circumference			
Yes	2 (4.8%)	2 (4.8%)	1^a^
No	40 (95.2%)	40 (95.2%)	
Tooth wear			
Yes	5 (11.9%)	3 (7.1%)	0.713^b^
No	37 (88.1%)	39 (92.9%)	
Pain			
Yes	23 (54.8%)	1 (2.4%)	<0.001^a^
No	19 (45.2%)	41 (97.6%)	
Large tongue size			
Yes	8 (19%)	4 (9.5%)	0.212^a^
No	34 (81%)	38 (90.5%)	
Bruxism			
Yes	12 (28.6%)	7 (16.7%)	0.192^a^
No	30 (71.4%)	35 (83.3%)	
Pressure teeth			
Yes	19 (45.2%)	8 (19%)	0.01^a^
No	23 (54.8%)	34 (81%)	
Hands under the chin			
Yes	6 (14.3%)	16 (38.1%)	0.013^a^
No	36 (85.7%)	26 (61.9%)	

^a^ Chi‐square test.

^b^ Fischer test.

In the case group, 11.9% and the control group, 9.5% (four people) had an object bite. The number of object bites between the two groups was not statistically significant (*p* = 1.00). 4.8% played musical instruments in the case group and 16.7% in the control group. There was no significant difference between the two groups in terms of playing a musical instrument (*p* = 0.156). 31% in the case group and 19% in the control group chewed gum. The number of people chewing gum in both groups was not statistically significant (*p* = 0.208). 33.3% in the case group and 40.5% in the control group had a habit of biting the cheek. The number of patients accustomed to cheek biting was not statistically significant in both groups (*p* = 0.498) (Table 4).

**Table 4:** Comparison of object biting, playing a musical instrument, chewing gum, and cheek biting in the control and case groups.

Variable	Case *n* (%)	Control *n* (%)	*p* Value
Object biting			
Yes	5 (11.9%)	4 (9.5%)	1^a^
No	37 (88.1%)	38 (90.5%)	
Playing a musical instrument			
Yes	2 (4.8%)	7 (16.7%)	0.156^b^
No	40 (95.2%)	35 (83.3%)	
Chew gum			
Yes	13 (31%)	8 (19%)	0.208^a^
No	29 (69%)	34 (81%)	
Cheek biting			
Yes	14 (33.3%)	17 (40.5%)	0.46^a^
No	28 (66.7%)	25 (59.5%)	

^a^ Chi‐square test.

^b^ Fischer test.

Table 5 shows the association of tongue size, pain, neck circumference, and gender variables with the *M* Score in the control and case groups. As seen in the case group, individuals with large tongue sizes and those with a neck circumference above 40 cm had significantly higher *M* scores (*p* = 0.047 and *p* = 0.017, respectively). The frequency distribution of *M* scores between people with pain and people without pain and between women and men was not statistically significant. In the control group, the frequency distribution of *M* Scores between people with large tongue size and normal people, between people with pain and people without pain, between people with a neck circumference of more than 40 cm with other individuals, and between women and men were not statistically significant (Table 5).

**Table 5:** Association of the variables of tongue size, pain, neck circumference, and gender with the *M* Score in the control and case groups.

Variable	Case *n* (%)				*p* Value^a^	Control *n* (%)				*p* Value^a^
	M1	M2	M3	M4		M1	M2	M3	M4	
Gender										
Male	7 (33.3%)	4 (19%)	8 (38.1%)	2 (9.5%)	0.453	17 (42.5%)	15 (37.5%)	8 (20%)	0 (0%)	0.055
female	4 (19%)	6 (28.6%)	7 (33.3%)	4 (19%)		12 (57.1%)	6 (28.6%)	3 (14.3%)	0 (0%)	
Tongue size										
Yes	0 (0%)	1 (12.5%)	6 (75%)	1 (12.5%)	0.047	3 (75%)	1 (25%)	0 (0%)	0 (0%)	0.176
No	11 (32.4%)	9 (26.5%)	9 (26.5%)	5 (14.7%)		14 (36.8%)	16 (42.1%)	8 (21.1%)	0 (0%)	
Pain										
Yes	7 (30.4%)	6 (26.1%)	6 (26.1%)	4 (17.4%)	0.591	1 (100%)	0 (0%)	0 (0%)	0 (0%)	0.595
No	4 (21.1%)	4 (21.1%)	9 (47.4%)	2 (10.5%)		16 (39%)	17 (41.5%)	8 (19.5%)	0 (0%)	
Neck circumference										
Yes	0 (0%)	0 (0%)	0 (0%)	2 (100%)	0.017	0 (0%)	2 (100%)	0 (0%)	0 (0%)	0.506
No	11 (27.5%)	10 (25%)	15 (37.5%)	4 (10%)		17 (42.5%)	15 (37.5%)	8 (20%)	0 (0%)	

^a^ Kendall's tau‐b.

The correlation between *M* Score with variables such as BMI, STOP‐BANG, and PSQI, and the amount of mouth opening in the case and control groups showed that in the case group, *M* Score with BMI (*p* = 0.006, *r* = 0.418) and PSQI (*p* = 0.007, *r* = 0.411) had a direct and significant relationship but with STOP‐BANG (*p* = 0.989, *r* = −0.002) and mouth opening rate (*p* = 0.355, *r* = −0.146) had a reverse relationship. Still, the correlation value was not significant for any of them.

## 4 DISCUSSION

TMD are among the diseases of the masticatory system. A high percentage of people in the community experience at least one of the symptoms of these disorders in their lifetime. Many side effects can be avoided with early diagnosis and simple prevention. The prevalence of TMD signs and symptoms has been reported in many different studies. On the other hand, several factors, such as social, personal, and even economic factors, as well as medical and dental history, can be involved in the development and prevalence of TMD (Martin et al., 2007).

Various studies have examined the prevalence of temporomandibular joint signs and symptoms in different communities. According to the research studies, TMD prevalence has been recorded in several populations ranging from 21.3% to 90% (Andrade et al., 2009). TMD patients are present in a wide age range. However, peak incidence has been reported between 20 and 40 years of age (Manfredini et al., 2011).

The disorder is 1.5−2 times more general in women than in men. Sexual superiority has been expressed in studies with different and contradictory results in further studies (Ferendiuk et al., 2014; Liljeström et al., 2008).

It is not yet clear why women are more prone to these disorders. In one study, a progressive symptom of temporomandibular disorder was seen in women taking oral contraceptives and women over 40 taking estrogen substitutes (Burket et al., 2003).

In 1983, Professor Mallampati proposed a method that made it possible to predict difficult intubation during the preoperative time. He tested the method in a double‐blind clinical trial in 1985 and considered three classes for airway evaluation (Mallampati et al., 1985). This test quickly became widespread due to its simplicity and efficiency. Butler and Dhara first proposed thyromental distance measurement, and its value in predicting the difficulty of intubation was investigated (Tabatabaian et al., 2013). In the present study, it was found that the Mallampati score of the TMD group was significantly about 1 point higher than the healthy group. No study has been done in this field so no comparison can be made.

In a study of 168 patients, Rodrigues et al. found a strong association between breath disorder during sleep and high Mallampati scores (Rodrigues et al., 2010). In line with this study, Liljestrom et al. stated that high Mallampati scores, if associated with nasal congestion, are strong predictors of OSA (Liljeström et al., 2008).

OSA is a chronic disorder marked by upper airway collapse during sleep. Various studies have also reported the relationship between temporomandibular joint dysfunction and OSA (Guilleminault, 2001; Tabatabaian et al., 2013).

The present study found that the maximum mouth opening was 47 mm in the control group and 43 mm in the case group, which is significantly lower in patients with TMD. In line with the present study, two other studies showed a significant association between TMD and maximum mouth opening (Choi et al., 2002; Deng et al., 2020). Contrary to the above study, AlHammad et al. stated that there was no significant relationship between TMD and maximal mouth opening. The result difference can be related to racial differences. So in the study of AlHammad et al., the maximum opening of the mouth is about 50 mm and is higher than in the present study (AlHammad et al., 2021).

Regarding the results of the STOP‐BANG questionnaire, it was found that the scores of this questionnaire did not differ significantly between the two groups. Also, the Pearson correlation coefficient showed no significant correlation between the Mallampati score and STOP‐BANG. Studies have shown that using the STOP‐BANG questionnaire + Mallampati score has better efficiency in screening. Avincsal et al. designed a study to improve the sensitivity and specificity of the STOP‐BANG questionnaire for OSA screening. They stated that the specificity of the modified STOP‐BANG questionnaire + the Mallampati score is more effective than the standard STOP‐BANG questionnaire in screening for sleep apnea (Avincsal et al., 2017).

Menon et al. in a cross‐sectional study, showed a significant agreement and relationship between the BMI index and Mallampati score (Menon et al., 2017). Other studies have shown that a high BMI, or obesity in general, increases laryngoscopic stiffness (Mallampati index above 3), which is in line with the results of the present study (De Cassai et al., 2020; Wang et al., 2018).

This study was conducted as a pilot due to the limited number of patients and available resources. The present study emphasizes that there is a significant relationship between the Mallampati score and TMD, nevertheless, it cannot be certainly said that this is a direct cause‐and‐effect relationship. The novelty of the present study and the lack of sufficient evidence in the literature make this issue more prominent or perhaps more ambiguous. The results of this study are noteworthy from the point of view that the Mallampati score can be used as an auxiliary clinical examination along with other clinical methods to be used as a simple diagnostic tool in the screening of TMD patients. Even if the accuracy of this tool is not high enough, it can be valuable to consider the high scores of this criterion in referring patients for further investigations.

One of the limitations of the present study is the sample size of 84 patients, which, this problem should be addressed in future studies. Another limitation of this study is the non‐use of more accurate clinical methods for diagnosing TMD, such as preparing CBCT of the patients' joints, which, due to the pilot nature of the study, will be on the agenda in future studies. Also, more accurate methods to evaluate the patients’ sleep quality and upper airway obstruction to find a more specific relationship between upper airway obstruction and TMD can be discussed in future studies.

## 5 CONCLUSION

The rate of mouth opening in the case group (with TMD problem) was significantly lower than in the control group. Also, joint pain has been seen more in the case group. The Mallampati score of the case group was significantly higher in the TMD group (about 1 point) than in the control group. Also, the score of the PSQI questionnaire was higher in the case group. Patients with larger tongues and larger necks had higher Mallampati scores. Pearson correlation coefficient showed that the Mallampati score had a direct and significant relationship with BMI and PSQI.

This study indicated that the Mallampati score could be used for the initial screening of TMD patients.

## AUTHOR CONTRIBUTIONS

**Amirtaher Mirmortazavi**: Conceptualization, resources, project administration. **Azam Sadat Madani**: Methodology, supervision, validation. **Saeed Hassanzadeh**: Investigation, original draft preparation. **Reza Shakiba**: Data analysis, review and editing.

## CONFLICT OF INTEREST STATEMENT

The authors declare no conflict of interest.

## Data Availability

The data sets analyzed during the current study are available from the corresponding author upon reasonable request. How to access the data can be obtained by requesting the corresponding author by email if necessary. Where this article contains proprietary or confidential data, the raw data cannot be made openly available. However, summary statistics derived from these data are available upon reasonable request.
